# Clinical Applications of Probiotics in Pediatric Dentistry and Orthodontics—A Systematic Review

**DOI:** 10.3390/nu17193153

**Published:** 2025-10-03

**Authors:** Lucia Giannini, Giovanna Stella, Giovanni Cattaneo, Gianna Dipalma, Cinzia Maspero

**Affiliations:** 1 Department of Biomedical, Surgical and Dental Sciences, University of Milan, 20122 Milan, Italy; lucia.giannini@unimi.it (L.G.); giovanna.stella@studenti.unimi.it (G.S.); gio.cat@hotmail.it (G.C.); gianna.dipalma@uniba.it (G.D.); 2 Fondazione IRCCS Cà Granda Ospedale Maggiore Policlinico, 20122 Milan, Italy; 3 Department of Interdisciplinary Medicine, University of Bari Aldo Moro, 70124 Bari, Italy

**Keywords:** probiotics, orthodontics, pediatric dentistry

## Abstract

Background: In recent years, scientific interest in probiotics in oral health has grown exponentially. This systematic review aims to analyze the effectiveness of probiotic use in dentistry, specifically in two areas: orthodontics and pediatric dentistry. Methods: Forty studies (RCTs, systematic reviews, clinical and preclinical studies) published between 2001 and 2025 were selected from the literature (PubMed) to evaluate the impact of probiotics on clinical, microbiological, and patient-specific parameters. Results: Results generally indicate that the most observed effect is the reduction in *Streptococcus mutans* levels, while in orthodontics, probiotics have proven beneficial primarily in reducing halitosis and traumatic lesions. In pediatric dentistry, early and prolonged use of probiotics has been shown to benefit both the reduction in caries and the improvement of gingival health, with significant results also observed in pediatric patients with special needs. Conclusions: Although the data obtained so far are very encouraging, further clinical studies are needed to define standardized protocols, identify the most effective strains, and evaluate the effects of long-term probiotic use. Probiotics therefore represent a promising and potentially valuable addition to preventive strategies in dentistry, particularly in orthodontics and pediatric dentistry, when integrated into a sustainable and personalized approach to patient oral health.

## 1. Introduction

The term “probiotic” derives from the Ancient Greek pro-bio, which literally means “for life [1].” This concept perfectly encapsulates its meaning: probiotics support and promote biological processes that contribute to the body’s well-being and promote its proper functioning [2]. The idea that microorganisms could exert beneficial effects is not a recent discovery: since the time of ancient civilizations, such as the Romans and Greeks, fermented foods (such as sour milk, primordial yogurts, and fermented beverages) have been used, attributing their healthful properties, albeit without a scientific explanation [1].

It was Élie Metchnikoff, a microbiologist and Nobel Prize winner in Physiology or Medicine in 1908, who laid the foundation for the scientific use of probiotics. Studying the longevity of some rural populations in Eastern Europe, Metchnikoff hypothesized that a high consumption of fermented milk rich in lactic acid bacteria could be linked to a reduced incidence of chronic diseases and a longer life expectancy. He hypothesized that the regular intake of “good” bacteria could counteract the growth of intestinal pathogens, and that this process could reduce intestinal putrefaction and the associated inflammatory processes [3]. This insight marked the beginning of a new era, in which diet and the microbiome were recognized as crucial to overall health.

With the advancement of microbiological and immunological knowledge throughout the 20th century, the concept of probiotics was progressively refined. Today, the World Health Organization (WHO) defines probiotics as “live microorganisms which, when administered in adequate amounts, confer a health benefit on the host” [2]. In recent decades, interest in these substances has extended well beyond the gastrointestinal tract, involving fields such as immunology, dermatology, and even oral health.

The oral cavity represents one of the most complex microbial ecosystems in the human body; it is populated by over 700 bacterial species that coexist in dynamic equilibrium. This balance, known as “oral microbiota homeostasis,” is essential for oral health and, indirectly, for systemic health [4]. Numerous scientific studies have demonstrated that alterations in the oral microbiome (known as dysbiosis) are associated not only with dental diseases such as caries, gingivitis, and periodontitis, but also with systemic diseases, such as cardiovascular disease, diabetes, and pregnancy complications [5].

Some of the most studied probiotic species are frequently found in human saliva and on mucosal surfaces; the most common include: *Lactobacillus acidophilus*, *Lactobacillus casei*, *Lactobacillus fermentum*, *Lactobacillus plantarum*, *Lactobacillus rhamnosus*, *Lactobacillus salivarius*, and *Weissella cibaria* [6].

These bacteria, due to their ability to compete with pathogens for attachment sites, produce antimicrobial substances, and modulate the local immune response, play a fundamental role in maintaining oral health and, consequently, systemic health [7].

When the microbial balance is compromised, for example, in patients with poor oral hygiene, a diet high in simple and complex sugars, prolonged antibiotic use, or orthodontic appliances, the risk of developing diseases such as caries, gingivitis, and periodontal disease increases significantly. In these contexts, the use of probiotics represents an innovative and biologically sustainable approach that can potentially complement traditional therapies, aiming to restore the physiological composition of the oral microbiota [8].

This review focuses on the role of probiotics in two specific fields:

- Orthodontic treatment: Patients with fixed appliances are prone to plaque accumulation and the growth of cariogenic microorganisms, which increase the risk of white spot lesions, caries, and gingivitis.
- Pediatric dentistry: Early modulation of the oral microbiome through probiotics may reduce caries prevalence and improve periodontal health in children and adolescents.

## 2. Materials and Methods

### 2.1. Protocol Employed

This systematic review was performed as per the PRISMA strategy and the rules from the Cochrane group.

Generative artificial intelligence (GenAI) was used in this article only to check and verify the table for errors.

This systematic review has been registered on PROSPERO (CRD420251140165).

### 2.2. Risk of Bias

The methodological quality of the randomized controlled trials (RCTs) included in this review was assessed using the Cochrane Collaboration’s Risk of Bias tool.

Each study was evaluated, and the overall risk of bias was categorized as low, some concerns, or high. The results of this assessment are summarized in Table 1.

### 2.3. Review Hypotheses

This review aimed to identify the current scientific evidence on the effectiveness of probiotics, examining the formulations used (specific bacterial strains, method of administration, duration of therapy), as well as the effects observed in pediatric and orthodontic patients. Furthermore, emphasis was placed on the future potential of probiotics as a support in preventing common complications during orthodontic treatment, such as gingivitis and oral dysbiosis, along with their role in modulating the oral microbial ecosystem in growing patients.

### 2.4. Study Selection

For this article, a systematic review was conducted by searching the PubMed engine; the MeSH terms ‘Dentistry’ [Mesh] AND ‘Probiotics’ [Mesh] were entered. From this search, 188 results emerged:

- 71 have periodontology as their topic;
- 15 have caries as a topic;
- 29 oral health in general;
- 17 implantology;
- 20 orthodontics;
- 6 prosthetics;
- 19 pedodontics;
- 1 endodontics;
- 4 on surgery;
- 1 on COVID;
- 1 smoking;
- 1 pregnancy;
- 2 dental materials;
- 1 article was not relevant to the research.

**Figure 1:**
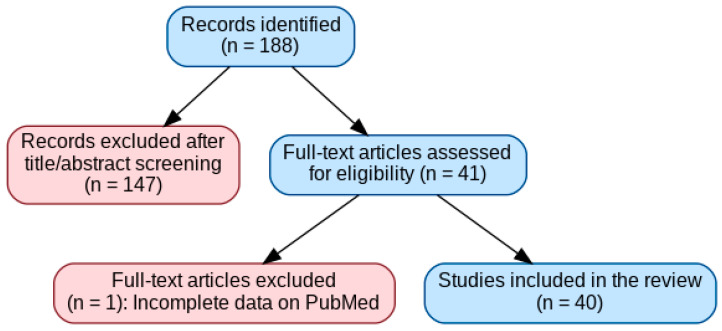
Flow Chart.

41 articles about pedodontics and orthodontics were selected, and finally one article out of 41 related to orthodontics was excluded as it was not entirely available on PubMed, for a total of 40 articles selected. All articles are dated between the year 2001 and 2025. As shown in Figure 1.

Randomized controlled trials (RCTs), systematic reviews, meta-analyses, in vitro studies, preclinical studies in animal models and pilot trials evaluating the effects of probiotics on various clinical and microbiological outcomes in orthodontically treated patients and pediatric patients were included in the review. Articles on other dental topics and articles not relevant to the research were excluded.

### 2.5. Inclusion Criteria

Randomised controlled trials (RCTs), systematic reviews, meta-analyses, in vitro studies, preclinical studies in animal models and pilot trials evaluating the effects of probiotics on various clinical and microbiological outcomes in orthodontically treated patients and pediatric patients were included in the review.

### 2.6. Exclusion Criteria

Articles on other dental topics and articles not relevant to the research were excluded. Articles not fully available on PubMed were also excluded.

For greater methodological transparency, we specified the inclusion and exclusion criteria a priori, and two independent reviewers reviewed all papers.

Study designs were then categorized (RCTs, systematic reviews, clinical trials, in vitro and animal studies) to allow for the stratification of evidence based on strength.

### 2.7. Search Strategy

Using the keywords “Dentistry” [Mesh] AND “Probiotics” [Mesh], a systematic search was conducted in the PubMed-MEDLINE database. Articles published between 2001 and 2025 were considered. The initial search yielded 188 results, of which 41 studies related to pediatric dentistry and orthodontics. Of these 41, one about orthodontics was excluded because the full article was not available on PubMed.

The 40 articles were included after applying inclusion and exclusion criteria. The search strategy aimed to identify randomized controlled trials (RCTs), systematic reviews, meta-analyses, in vitro studies, and preclinical animal studies evaluating the effects of probiotics on oral health, specifically in orthodontic patients and children.

### 2.8. Data Selection and Coding

Two independent reviewers selected and compared the articles. Subsequently, the two reviewers created two separate tables: one for articles in the field of orthodontics (Table 2) and one for articles in the field of pediatric dentistry (Table 3). The following data were extracted from each table: authors, title, year, study design, participants, and results.

### 2.9. Statistical Analysis

Due to the heterogeneity in study design, probiotic strains, and administration methods, a qualitative synthesis was prioritized over a comprehensive quantitative meta-analysis. Where meta-analyses were available within the included systematic reviews, effect sizes and confidence intervals were analyzed. Descriptive statistics were used to summarize the distribution of studies by type of intervention and outcome.

## 3. Results

The analysis of the 40 studies allowed us to outline a complete picture of the role of probiotics in orthodontics and pediatric dentistry. The heterogeneity of the strains used, the methods of administration and the experimental designs represented a challenge, but also an opportunity to evaluate the multifactorial impact of probiotics in the oral cavity.

- Types of Included Studies

This systematic review included a total of 40 studies addressing the role of probiotics in orthodontics and pediatric dentistry. These studies employed various research designs, reflecting the current diversity of research approaches in this field. The distribution of studies by type is as follows:

- *Randomized controlled trials (RCTs): 11 studies.* These represent the highest level of evidence among the included studies and investigated the efficacy of probiotics under controlled clinical conditions.
- *Systematic reviews: 6 studies;* These provided a comprehensive synthesis of the available evidence, assessing the overall effect of probiotics on oral health.
- *Meta-analyses: 2 studies.* These studies combined data from multiple randomized clinical trials to provide an overall quantitative estimate of the effects of probiotics on oral health.
- *Clinical trials (non-RCTs): 9 studies.* These evaluated probiotics without complete randomization.
- *Pilot studies: 4 studies.* Conducted primarily to evaluate feasibility, these studies provided preliminary data on clinical benefits and tolerability.
- In vitro *studies: 4 studies.* These evaluated the antimicrobial effects of probiotics and their interaction with orthodontic materials.
- *Preclinical/animal studies: 3 studies.* Conducted in animal models, these explored mechanistic aspects, such as the impact on bone remodeling during orthodontic treatment.
- *Narrative review: 1 study.* A narrative review addressed the potential use of *Streptococcus* spp. for the prevention of caries in patients with special needs.

- Setting of Included studies

Clinical trials and RCTs were mostly conducted in academic or hospital-based orthodontic and pediatric dental clinics. Pilot studies and clinical studies were performed in outpatient practices or university settings. In vitro and animal studies were carried out in controlled laboratory conditions. The geographic distribution covered multiple countries, including the USA, European countries and regions in Asia and South America.

- Area of the Mouth Assessed

Studies have examined different areas of the oral cavity depending on their objective: Orthodontic studies have typically assessed plaque accumulation around orthodontic brackets, the development of white spot lesions, and gingival health in patients with fixed appliances. Pediatric dentistry studies have focused on saliva and plaque samples for microbiological analysis (e.g., *Streptococcus mutans*, *Lactobacillus counts*), as well as the development of caries in primary and permanent teeth in general.

- Comparisons Used in Included Studies

The included studies compared different varieties of probiotics and formulations.

Different probiotic formulations (toothpaste, lozenges, chewable tablets, yogurt, skim milk, mouthwash) were compared to placebo or conventional products.

In orthodontic studies, probiotic-enriched products were compared to standard fluoride toothpaste or placebo lozenges. In pediatric studies, probiotic supplementation was compared to a regular diet, fluoride toothpaste, or placebo tablets. In vitro and animal studies compared exposure to probiotics with control groups without probiotics to evaluate antimicrobial effects and interactions with orthodontic materials.

- Results of Included Studies

The outcomes evaluated varied depending on the different types of studies. We can classify the different types of outcomes as follows:

- Microbiological outcomes: Some studies evaluated changes in the counts of *Streptococcus mutans*, *Lactobacillus*, and other bacterial strains in saliva or plaque.
- Clinical outcomes: Some studies evaluated the plaque index, gingival bleeding index, and the development of white spot lesions.
- Patient-focused outcomes: Other studies evaluated the potential reduction in halitosis, potential improvement of traumatic lesions in orthodontic patients, and changes in salivary pH.
- Material-related outcomes: In vitro studies evaluated the effect of probiotics on the surface roughness and hardness of orthodontic alloys.
- Bone-related outcomes: Animal studies evaluated the speed of orthodontic tooth movement and osteoclast activity.

- Methodological Quality of Included Studies

Methodological quality varied by study type, with systematic reviews and meta-analyses generally of moderate quality, inconsistently reported dropout rates, and inherent limitations in extrapolation to clinical practice in the case of in vitro and preclinical studies, which nevertheless provided useful mechanistic insights.

1. ***Probiotics and orthodontics***

Several studies have documented a significant reduction in the load of *Streptococcus mutans* in patients with fixed orthodontic appliances, suggesting a preventive effect against caries. The meta-analyses of Robin et al. [20] and Chen et al. [21] confirm this data. Clinical studies, such as that of Alp et al. [23], have shown a reduction in *Streptococcus mutans* and *Lactobacillus* spp. with the use of kefir and probiotic toothpastes, accompanied by an increased buffering capacity of saliva.

Benic et al. [10] observed a significant reduction in halitosis following the intake of *Streptococcus salivarius M18*. Silva et al. [12] instead documented a decrease in the duration and intensity of traumatic lesions from braces thanks to the use of *Lactobacillus brevis CD2*. In 2025, Tahir et al. [13] found an improvement in the gingival bleeding index through the use of toothpastes containing probiotics. However, not all studies agree. Gizani et al. [11] and Hadj-Hamou et al. [24] did not find significant effects in the prevention of white spot lesions.

In vitro studies (Pavlic et al. [22]; Lemos et al. [26] have shown that probiotics can modify metal surfaces, increasing roughness and reducing microhardness, with possible implications on orthodontic materials. Finally, preclinical studies in animals (Duffles et al., 2022 [27]; Pazzini et al., 2017 [28]) suggest that *Bifidobacterium animalis* and *Bacillus subtilis* can slow tooth movement by reducing osteoclastic activity.

2. ***Probiotics and Pediatric Dentistry***

In pediatrics, probiotics have been shown to be effective in reducing *Streptococcus mutans* and improving gingival health. The meta-analysis conducted by Mayta-Tovalino et al. in 2024 [37] confirmed a reduction in *Streptococcus mutans* values in over 2600 patients.

Longitudinal studies (Stensson et al. [16]) indicate that the administration of *L. reuteri* in the first year of life is associated with fewer caries and gingivitis at 9 years of age. Clinical studies (Jindal et al. [40]; Alamoudi et al. [15]) confirm the reduction in *Streptococcus mutans* in children treated with probiotics. Formulations such as candies and lozenges (Hedayati-Hajikand et al. [14]; Kavitha et al. [38]) have shown benefits even in short-term treatments.

Sujlana et al. [34] observed clinical improvements in children with special needs, through the use of mouthwashes containing probiotics in subjects with hearing impairment. Mato et al., in 2024, studied the use of antagonistic strains of *Streptococcus mutans* in patients with neuromotor disabilities [35].

Finally, Lexner et al. [42] and Taipale et al. [43,44] demonstrated that milk and supplements containing *L. rhamnosus* and *B. lactis* can significantly reduce early colonization by *Streptococcus mutans*, although an impact on long-term caries was not always found.

## 4. Discussion

Over the past two decades, there has been growing attention to the role of probiotics in the health of the human organism and in the health of the oral cavity. Advances in microbiology have highlighted the importance of maintaining oral microbial homeostasis, which is essential not only for oral health but also for overall systemic well-being. The possibility of restoring this balance in a biologically sustainable manner through beneficial live microorganisms has opened new perspectives in dental practice, particularly in two sensitive and complex areas: orthodontics and pediatric dentistry.

This review has shown that the use of probiotics in orthodontics presents a promising strategy from both clinical and microbiological perspectives. Patients who benefit from fixed orthodontic treatment present an altered oral environment, more vulnerable to plaque accumulation and the proliferation of cariogenic microorganisms, which makes this population particularly interesting for the study of the efficacy of probiotics.

One of the major challenges that emerged during this review is the marked heterogeneity of study designs, probiotic strains, administration routes, and follow-up times. This variability explains the inconsistencies between studies and limits the ability to draw definitive conclusions, especially when comparing clinical and microbiological results.

A limitation of our review is the heterogeneity of the included studies.

To avoid different levels of evidence, we have stratified our conclusions according to study type. The most consistent and clinically relevant findings are the ones from RCTs and systematic reviews, which confirm the ability of probiotics to reduce *Streptococcus mutans* counts and to improve some gingival health indices in both pediatric and orthodontic patients.

Randomized clinical trials support the halitosis reduction and the attenuation of traumatic lesions.

In contrast, findings from in vitro studies (probiotic interactions with orthodontic alloys) and from preclinical animal models (effects on osteoclast activity or orthodontic alloys) provide valuable insights but cannot be directly extrapolated to clinical practice.

However, in vitro and animal studies were included in this review to provide a broader and more complete picture of the field. While these studies cannot replace clinical evidence, they offer important mechanistic insights that are difficult to obtain in the clinical setting, such as the interaction of probiotics with orthodontic materials or their potential influence on bone remodeling. For this reason, while acknowledging their inherent limitations, we stratified the results by study type, emphasizing that RCTs and systematic reviews represent the most robust and reliable evidence for clinical applications, while preclinical studies should be considered primarily for hypothesis generation and to support clinical observations.

In this way, pilot studies and narrative reviews should be interpreted with caution.

This funding suggests that the strongest clinical applications are supported by evidence from randomized controlled trials, while mechanistic or early findings still need more validation before they can be applied in pediatric dentistry and orthodontics.

One of the most important results is the significant reduction in the bacterial load of *Streptococcus mutans*, the main bacterium implicated in the genesis of caries. This reduction has been documented in meta-analyses (Robin et al. in 2025 [20]; Chen et al. in 2023 [21]) and in controlled clinical trials, confirming the potential role of probiotics as adjuvants in the prevention of carious lesions during orthodontic treatment.

Alp et al. [23] reported that kefir and probiotic toothpaste slightly reduced salivary concentrations of *Streptococcus mutans* and *Lactobacillus* spp. and increased salivary buffering capacity, suggesting improved resistance to acidogenic bacterial challenges. Benic et al. [10] found that *Streptococcus salivarius M18* significantly reduced halitosis in orthodontic patients, thereby improving quality of life.

Silva et al. [12] then demonstrated a reduction in the duration and intensity of traumatic lesions caused by orthodontic appliances through the administration of *Lactobacillus brevis CD2*, suggesting an anti-inflammatory and soothing action by probiotics on the oral mucosa. An improvement in periodontal parameters, such as the gingival blood index, has also been observed in studies such as that of Tahir et al., in 2025, where the use of probiotic toothpastes led to a significant decrease without, however, significantly modifying the plaque index [13].

However, not all studies agree on the clinical efficacy of probiotics, not finding significant preventive effects on the appearance of white spot lesions associated with orthodontic therapy (Gizani et al. [11] and Hadj-Hamou et al. [24]).

It is important to note that, particularly in orthodontics, reductions in microbial markers such as *Streptococcus mutans* do not always correspond to clear clinical benefits; this discrepancy may reflect the multifactorial etiology of and white spot lesions, in which host, diet, oral hygiene, and treatment duration factors play a decisive role. Furthermore, the heterogeneity of probiotic strains, formulations, and study protocols, along with the relatively short follow-up in many studies, may limit the translation of microbiological improvements into consistent clinical outcomes.

Several factors may explain this lack of correspondence between microbiological improvements and clinical endpoints. First, many of the available studies have relatively short follow-up periods and are therefore likely insufficient to detect changes in caries incidence or the development of white spot lesions. Second, the effects of probiotics are often strain-specific, and heterogeneity in strains and formulations may explain inconsistent results. Patient compliance with daily intake of probiotics in various formulations also plays a key role. These factors therefore suggest that microbiological changes alone cannot be considered a guarantee of clinical benefits, reinforcing the need for long-term, well-controlled studies with standardized protocols.

From the microbiological and biomaterials point of view, another important aspect has emerged: the interaction between probiotics and orthodontic materials. In vitro studies (Pavlic et al. [22]; Lemos et al. [26]) have shown that prolonged exposure to probiotics can modify the characteristics of metal surfaces, increase their roughness and reducing their microhardness, especially in steel and titanium alloys.

Finally, preclinical studies conducted on animal models (Duffles et al. in 2022 [27] and Pazzini et al. in 2017 [28]) have suggested an action of probiotics on bone metabolism: the administration of strains such as *Bifidobacterium animalis* and *Bacillus subtilis* was associated with a reduction in the number of osteoclasts and a slowing of tooth movement, indicating a possible role in the regulation of bone remodeling induced by orthodontic therapy.

In summary, the results obtained suggest that the use of probiotics in orthodontics can bring concrete benefits ranging from the modulation of the oral microbiota to a reduction in symptoms associated with the treatment, including the possible effects on bone tissue and materials used. While certain aspects require further clarification, overall findings are encouraging and support the integration of probiotics into modern orthodontic practice.

In pediatric dentistry, the results appear even more promising. Children and adolescents, for both anatomical and behavioral reasons, have a high risk of caries and gum disease. An early intervention on the oral microbiota therefore represents a crucial opportunity in a preventive perspective.

The meta-analysis by Mayta-Tovalino et al. (2024), including 19 randomized clinical trials involving a total of more than 2600 patients, clearly demonstrates the efficacy of probiotics in reducing the load of *Streptococcus mutans* [37].

It is also important to note that some longitudinal studies have found long-term benefits. For example, Stensson et al. [16] showed that the administration of *Lactobacillus reuteri* in the first year of life can lead to a lower prevalence of caries and gingival inflammation at 9 years of age.

Numerous clinical studies, such as Alamoudi et al. [15] show a significant reduction in the load of *Streptococcus mutans* in children treated with probiotics. The formulations used range from common yogurt to sweets, such as the probiotic candies and tablets analyzed by Kavitha et al. in 2022 [38] and Hedayati-Hajikand et al. in a study of 2015, which showed significant improvements in the plaque index and gingival health even in short-term treatments [14].

Particularly significant are the studies that evaluate the use of probiotics in pediatric populations with special needs. For example, Sujlana et al. [34] demonstrate clinical improvement in children with hearing impairment with the use of probiotic mouthwashes, while Mato et al. in 2024 explore the potential of *Streptococcus* strains antagonistic to *Streptococcus mutans*, useful in patients with cognitive or neuromotor disabilities [35].

These results, while encouraging, must be interpreted with caution. Many long-term studies suffer from sample attrition, which can introduce bias into the results, and most were conducted in specific geographical or cultural contexts, limiting their generalizability. Similarly, studies on children with special needs, while highly relevant, often involve small and heterogeneous samples, making it difficult to extend the conclusions to the broader pediatric population. Replication in larger and more diverse cohorts is therefore necessary before these findings can be translated into routine clinical practice.

It has also been shown that the use of milk and supplements containing strains such as Lactobacillus *rhamnosus* and *Bifidobacterium lact* is cause a reduction in early colonization by oral pathogens [42,43,44]. However, some of these studies suggest that the long-term benefit of probiotics in preventing caries could also depend on concomitant environmental, nutritional and behavioral factors.

In order to strengthen the transparency of our review, we performed a formal risk-of-bias assessment for the included RCTs using the Cochrane Collaboration tool.

This evaluation showed that most studies presented a low or moderate risk of bias, while a few had methodological limitations. These aspects should be considered when interpreting our findings.

### Publication Bias and Sponsorship Considerations

It is important to acknowledge that some of the included studies evaluated commercial probiotic formulations. However, potential conflicts of interest and sponsorship were not always explicitly reported. This raises the possibility of publication bias, as industry- funded studies may be more likely to report favorable outcomes. Therefore, the positive findings observed in some trials should be interpreted with caution, and future research should ensure transparent disclosure of funding sources and potential conflicts of interest to strengthen the reliability of the evidence.

## 5. Conclusions

Based on the available evidence, some considerations can be drawn. First, it seems that the most consistent and replicable benefit of probiotic use concerns the reduction in *Streptococcus mutans* levels. This result is observable both in the sample of pediatric patients and in orthodontic patients. The clinical effects on plaque, gingivitis and caries probably depend on the type of probiotic strain used, the formulation (local or systemic), the duration of treatment and the target population.

In orthodontics, probiotics may offer secondary benefits such as reduced halitosis, fewer traumatic lesions, and improved gingival health more than direct caries prevention. Potential interactions with orthodontic materials and effects on tooth movement also warrant further investigation.

In pediatric dentistry, probiotics seem to represent a promising strategy, especially at an early age, in children at risk and in patients with special needs. Long-term and targeted formulations may enhance their preventive potential.

In conclusion, the efficacy of probiotics seems more marked in pediatric age and is also found in children with special needs or with conditions predisposing to caries.

The most consistent and replicable effect of probiotics across age groups and clinical settings is the reduction in *Streptococcus mutans* levels.

The prevention of caries and white spot lesions is currently not sufficiently supported by solid clinical evidence, but some longitudinal studies show promising results.

The most reliable evidence comes from RCTs and systematic reviews, confirming the reduction in *Streptococcus mutans* and some gingival benefits.

Findings from in vitro and animal studies cannot be directly translated to clinical practice.

Probiotics show promise for pediatric dentistry and orthodontics, but their routine clinical use awaits confirmation from multicenter randomized controlled trials with standardized protocols [45,46].

Overall, the most robust and replicable results, supported by RCTs and systematic reviews, concern the reduction in *Streptococcus mutans* and some improvements in gingival indices. Regarding in vitro and animal studies, however, while valuable for understanding the mechanisms, they should not be considered directly translatable into clinical practice. This distinction is essential to guide future research and avoid overinterpretation of initial findings.

In conclusion, although probiotics cannot yet be considered a universal solution for oral diseases, they represent a potentially useful tool. Their integration into clinical practice may be especially valuable in pediatric dentistry and supportive orthodontic care. Further research is essential to identify the most effective strains, refine treatment protocols, and confirm their long-term clinical impact.

In this regard, we emphasize that the aim of this review is not to provide definitive answers, but rather to synthesize the current state of evidence, highlight promising avenues, and outline the need for more standardized and strain-specific studies to refine our understanding.

## Figures and Tables

**Table 1:** Risk-of-bias was assessed according to the Cochrane Collaboration tool. Studies were classified as low, some concerns, or high risk of bias depending on the presence of methodological limitations.

Topic	Authors (Year)	Study Design	Random Sequence Generation	Allocation Concealment	Blinding (Participants/Personnel)	Blinding (Outcome Assessment)	Incomplete Outcome Data	Selective Reporting	Overall Risk of Bias
Orthodontics	Ritthagol W. et al. (2014) [9]	Double-blind RCT (cleft patients)	Low	Low	Low	Low	Low	Low	Low
Orthodontics	Benic G.Z. et al. (2019) [10]	Triple-blind RCT	Low	Low	Low	Low	Low	Low	Low
Orthodontics	Gizani S. et al. (2016) [11]	Double-blind RCT	Low	Low	Low	Low	Low	Low	Low
Orthodontics	Silva N.L.N.V. et al. (2021) [12]	Phase 2 RCT	Unclear	Low	Low	Low	Low	Low	Some concerns
Orthodontics	Tahir K. et al. (2025) [13]	Open-label RCT	Low	Low	High	High	Low	Low	High
Pediatric Dentistry	Hedayati-Hajikand T. et al. (2015) [14]	RCT	Low	Low	Low	Low	Low	Low	Low
Pediatric Dentistry	Alamoudi N.M. et al. (2018) [15]	RCT	Low	Low	Low	Low	Low	Low	Low
Pediatric Dentistry	Stensson M. et al. (2014) [16]	Single-blind, placebo-controlled study	Low	Low	Low	Low	Some concerns (attrition)	Low	Some concerns
Pediatric Dentistry/Orthodontics	Kaklamanos E.G. et al. (2019) [17]	RCT protocol	Low	Low	Low	Low	Not applicable	Not applicable	Low
Pediatric Dentistry	Stecksén-Blicks C. et al. (2009) [18]	Cluster RCT	Low	Low	High	High	Some concerns	Low	Some concerns
Pediatric Dentistry	Näse L. et al. (2001) [19]	RCT	Low	Low	Low	Low	Some concerns (attrition)	Low	Some concerns

**Table 2:** The table shows the data collected from the Orthodontics articles: authors, title, year, study design, participants, and results.

Authors	Title	Year	Study Design	Participants (Number, Age, Gender)	Outcomes
Robin V, Wim T, Maria CL, Isabelle L.	Probiotics for maintaining oral health during fixed orthodontic treatment: A systematic review and meta-analysis [20].	2025	Systematic review and meta-analysis	At least 10 patients/group undergoing fixed orthodontic therapy	Reduction in *Streptococcus mutans* counts, no significant effect on lactobacilli and white spot lesions. Interesting results on halitosis and oral traumatic lesions. Equivocal effects on plaque.
Chen W, Ren J, Li J, Peng S, Zhang C, Lin Y.	Effects of probiotics on the oral health of patients undergoing orthodontic treatment: a systematic review and meta-analysis [21].	2023	Systematic review and meta-analysis	405 Patients undergoing orthodontic treatment	Controversial results on clinical outcomes (plaque, gingival index). Significant reduction in *Streptococcus mutans*.
Pavlic A, Perissinotto F, Turco G, Contardo L, Spalj S.	Do Chlorhexidine and Probiotics Solutions Provoke Corrosion of Orthodontic Mini-implants? An In Vitro Study [22].	2019	In vitro study	0	Probiotics increase the roughness of titanium implants, while chlorhexidine increases the roughness of steel implants. Chlorhexidine combined with probiotics reduces the microhardness of steel implants.
Alp S, Baka ZM.	Effects of probiotics on salivary Streptecoccus mutans and Lactobacillus levels in orthodontic patients [23].	2018	Clinical study	45 orthodontic patients: (27 F, 18 M) (3 groups of 15: 15 patients in the kefir group (7 girls, 8 boys; mean age, 14.3 ± 1.7 years), 15 patients in the dentifrice group (10 girls, 5 boys; mean age, 14.9 ± 2.0 years), and 15 patients in the control group (10 girls, 5 boys; mean age, 14.1 ± 2.1 years).	Significant reduction in *Streptococcus mutans* and Lactobacillus with the use of probiotics (kefir and toothpaste). Significant increase in buffering capacity with the use of probiotic-containing toothpaste.
Hadj-Hamou R, Senok AC, Athanasiou AE, Kaklamanos EG.	Do probiotics promote oral health during orthodontic treatment with fixed appliances? A systematic review [24].	2020	systematic review	0	No significant effect of probiotics on gingival inflammation and enamel demineralization.
Pietri FK, Rossouw PE, Javed F, Michelogiannakis D.	Role of Probiotics in Oral Health Maintenance Among Patients Undergoing Fixed Orthodontic Therapy: a Systematic Review of Randomized Controlled Clinical Trials [25].	2020	Systematic Review of Randomized Controlled Clinical Trials	The total number of participants across all included studies ranged between 24 and 85. In 8 studies, the patients’ ages ranged from 8 to 35 years. Six studies included both male and female participants.	Improvement of oral health and reduction in pathogenic bacteria with probiotics. Conflicting results on plaque and gingivitis. No effect on white spot lesions formation.
Ritthagol W, Saetang C, Teanpaisan R.	Effect of Probiotics Containing *Lactobacillus paracasei* SD1 on Salivary Mutans Streptococci and Lactobacilli in Orthodontic Cleft Patients: A Double-Blinded, Randomized, Placebo-Controlled Study [9].	2014	Double-blinded, randomized, placebo-controlled study	30 orthodontic patients with cleft lip and palate (mean age 19.22 ± 3.66 years)	Significant reduction in mutans *streptococci* and increase in lactobacilli with *L. paracasei* SD1.
Lemos IDS, Jassé FFA, Suzuki SS, Alencar CM, Fujii DN, Zaniboni JF, Suzuki H, Garcez Segundo AS.	Antimicrobial activity of probiotics against oral pathogens around orthodontic mini-implants: an in vitro study [26].	2021	In vitro study	120 mini-screw	L. casei, L. brevis, L. rhamnosus and Lactobacillus from fermented milk Batavito^®^ showed antimicrobial activity against S. aureus.
Duffles LF, Menino AP, Taira TM, de Oliveira S, Salvador SL, Messora MR, Vinolo MAR, Fukada SY.	Probiotic *Bifidobacterium animalis* subsp. lactis consumption slows down orthodontic tooth movement in mice [27].	2022	Clinical study	30 male C57BL6/J mice	In mice, *Bifidobacterium animalis* subsp. lactis slowed orthodontic tooth movement.
Benic GZ, Farella M, Morgan XC, Viswam J, Heng NC, Cannon RD, Mei L.	Oral probiotics reduce halitosis in patients wearing orthodontic braces: a randomized, triple-blind, placebo-controlled trial [10].	2019	Randomized, triple-blind, placebo-controlled trial	64 patients with fixed orthodontic appliances (32 in the probiotic group, 32 in the placebo group)	Reduction in halitosis with *Streptococcus salivarius* M18. Mild effects on plaque and gingivitis.
Silva NLNV, Della Bona A, Cardoso M, Callegari-Jacques SM, Fornari F.	Lactobacillus brevis CD2 attenuates traumatic oral lesions induced by fixed orthodontic appliance: A randomized phase 2 trial [12].	2021	randomized phase 2 trial	20 orthodontic patients (14–57 years, 70% women in the probiotic group, 60% women in the placebo group)	The use of Lactobacillus brevis CD2 reduced the duration of traumatic oral lesions and pain.
Tahir K, Barakaat AA, Shoukat Ali U, Fida M, Sukhia RH.	Effect of probiotic toothpaste and regular toothpaste on gingival health and plaque levels of adult orthodontic patients—An open label randomized controlled trial [13].	2025	Open label randomized controlled trial	44 adult orthodontic patients (18–50 years)	Probiotic toothpaste improved the gingival bleeding index, but had no effect in the plaque index.
Gizani S, Petsi G, Twetman S, Caroni C, Makou M, Papagianoulis L.	Effect of the probiotic bacterium *Lactobacillus reuteri* on white spot lesion development in orthodontic patients [11].	2016	Randomized double-blind placebo-controlled study	85 patients (average age15.9 years)	Probiotic intake did not influence the development of white spot lesions.
Pazzini CA, Pereira LJ, da Silva TA, Montalvany-Antonucci CC, Macari S, Marques LS, de Paiva SM.	Probiotic consumption decreases the number of osteoclasts during orthodontic movement in mice [28].	2017		Mice C57BL/6	In mice, Bacillus Sub-ti-is was associated with a reduction in osteoclasts during orthodontic movement.
Kolip D, Yılmaz N, Gökkaya B, Kulan P, Kargul B, MacDonald KW, Cadieux PA, Burton JP, James KM.	Efficacy of Dentaq^®^ Oral and ENT Health Probiotic Complex on Clinical Parameters of Gingivitis in Patients Undergoing Fixed Orthodontic Treatment: A Pilot Study [29].	2016	Pilot study	15 patients (11–18 years)	Probiotics reduced plaque and gingival inflammation.
Jose JE, Padmanabhan S, Chitharanjan AB.	Systemic consumption of probiotic curd and use of probiotic toothpaste to reduce *Streptococcus mutans* in plaque around orthodontic brackets [30].	2013	Clinical study	60 orthodontic patients divided into 3 groups of 20: Group 1: control. Group 2: probiotic yogurt, Group 3: were asked to brush their teeth twice a day with a probiotic toothpaste (GD toothpaste).	Probiotic curd and probiotic toothpaste reduced *Streptococcus mutans* in plaque.
Gokce G, Savas S, Kucukyilmaz E, Veli I.	Effects of toothpastes on white spot lesions around orthodontic brackets using quantitative light-induced fluorescence (QLF): An in vitro study [31].	2017	In vitro study	45 extracted mandibular first molars in which caries were artificially recreated.	Significant differences between the initial and post-treatment QLF measurements of the demineralized enamel samples treated with the various agents (*p* < 0.05). In all experimental groups, a significant increase in fluorescence radiance and a decrease in lesion area were found (*p* = 0.000).
Voronkova AV, Smaglyuk LV.	Changes in biochemical parameters of oral fluid in patients during the orthodontic treatment with a bracket system under the action of a developed mucosal gel with probiotic [32].	2018	Clinical study	45 patients (18–24 years)	The gel with probiotics improved the biochemical parameters of the oral fluid.
Cildir SK, Germec D, Sandalli N, Ozdemir FI, Arun T, Twetman S, Caglar E.	Reduction of salivary mutans streptococci in orthodontic patients during daily consumption of yoghurt containing probiotic bacteria [33].	2009	Double-blind, randomized crossover study	24 adolescents (12–16 years)	Yogurt with *Bifidobacterium animalis* subsp. *lactis* DN-173010 reduced *mutans streptococci*.

**Table 3:** The table shows the data collected from the Pedodontics articles: authors, title, year, study design, participants, and results.

Authors	Title	Year	Study Design	Participants (Number, Age, Gender)	Outcomes
Sujlana A, Goyal R, Pannu P, Opal S, Bansal P.	Visual pedagogy and probiotics for hearing impaired children: A pilot study [34].	2017	Pilot study	20 children with hearing problems and 20 healthy children	Probiotic mouthwash reduced GI and PI and increased salivary pH.
Mato EG, Montaño-Barrientos BJ, Rivas-Mundiña B, Aneiros IV, López LS, Posse JL, Lamas LM.	Anti-caries *Streptococcus* spp.: A potential preventive tool for special needs patients [35].	2024	Narrative review	The text does not report data relating to a specific sample (number of patients), nor information on the sex or age of the participants.	Review of *Streptococcus* spp. with anti-*Streptococcus mutans* activity.
Cortés-Dorantes N, Ruiz-Rodríguez MS, Karakowsky-Kleiman L, Garrocho-Rangel JA, Sánchez-Vargas LO, Pozos-Guillén AJ.	Probiotics and their effect on oral bacteria count in children: a pilot study [36].	2015	Pilot study	40 patients (4–6 years) at high risk of caries	Probiotics reduced microbial count.
Mayta-Tovalino F, Maguiña-Quispe J, Barja-Ore J, Hernandez AV.	Efficacy of Probiotic Consumption on Oral Outcomes in Children and/or Adolescents: A Meta-Analysis [37].	2024	Meta-analysis	2622 between children and adolescents	Probiotics had no effect on caries reduction or *Lactobacillus* count. Positive effect on *Streptococcus mutans* count.
Kavitha M, Prathima GS, Anusha D, Kengadaran S, Gayathri K, Vinothini V.	Evaluation of the efficacy of plaque reduction and gingival health among 6–12 years old school children before and after a short term daily intake of probiotic lozenge—A comparative study [38].	2022	Comparative study	6–12 years	The group taking probiotics showed a statistically significant reduction in plaque indices compared to the placebo group, and a significant improvement in gingival health was also observed.
Borrell García C, Ribelles Llop M, García Esparza MÁ, Flichy-Fernández AJ, Marqués Martínez L, Izquierdo Fort R.	The use of *Lactobacillus reuteri* DSM 17938 and ATCC PTA 5289 on oral health indexes in a school population: A pilot randomized clinical trial [39].	2021	pilot randomized clinical trial.	27 teenagers, 12–18 years old	Probiotics reduced *Streptococcus mutans* in saliva and plaque.
Hedayati-Hajikand T, Lundberg U, Eldh C, Twetman S.	Effect of probiotic chewing tablets on early childhood caries—a randomized controlled trial. BMC Oral Health [14].	2015	RCT	138 children aged 2–3 years	The early development of childhood caries could be reduced through the administration of probiotic chewing tablets as an adjunct to the daily use of fluoride toothpaste in preschool children.
Jindal et al.	A comparative evaluation of probiotics on salivary mutans streptococci counts in Indian children [40]	2011	Clinical study	150 healthy children aged 7–14 years	Statistically significant reduction in salivary *Streptococcus mutans* counts in groups taking probiotics
Alamoudi NM et al.	Effect of probiotic *Lactobacillus reuteri* on Salivary Cariogenic Bacterial Counts among Grounp of Preschool Children in Jeddah, Saudi Arabia: A Randomized Clinical Trial [15]	2018	RCT	178 healthy children (3–6 years)	Probiotics containing L. reuteri significantly reduce cario-associated bacterial counts.
Stensson M et al.	Oral administration of *Lactobacillus reuteri* during the first year of life reduces caries prevalence in the primary dentition at 9 years of age [16].	2014	Single-center, single-blind, placebo-controlled study	Children (1 year of age followed up to 9 years) N = 113 (60 Probiotic, 53 Placebo)	Reduced caries prevalence, lower prevalence of approximal carious lesions, fewer sites with gingivitis in the probiotic group.
Cannon M, et al.	Effectiveness of CRT at measuring the salivary level of bacteria in caries prone children with probiotic therapy [41].	2013	Clinical study	60 children 6–12 years old	Statistically significant difference in CRT results between pre- and post-use of probiotics.
Kaklamanos EG et al.	A single-centre investigator-blinded randomised parallel group clinical trial to investigate the effect of probiotic strains *Streptococcus salivarius* M18 and *Lactobacillus acidophilus* on gingival health of pediatric patients undergoing treatment with fixed orthodontic appliances: study protocol [17].	2019	RCT	Pediatric patients undergoing orthodontic treatment N = 50	Reduction in gingival bleeding. Reduction in plaque and gingival indices, change in oral microbiome composition.
Lexner MO et al.	Microbiological profiles in saliva and supragingival plaque from caries-active adolescents before and after a short-term daily intake of milk supplemented with probiotic bacteria—a pilot study [42].	2010	Randomized, double-blind, placebo-controlled pilot study	Adolescents (with active caries) N = 18	No statistically significant difference in microbial profiles or levels of caries-associated bacteria.
Stecksén-Blicks C et al.	Effect of long-term consumption of milk supplemented with probiotic lactobacilli and fluoride on dental caries and general health in preschool children: a cluster-randomized study [18].	2009	Cluster randomized study	Preschool children (1–5 years) N = 248	Reduced caries increment, fewer days of antibiotic therapy.
Näse L et al.	Effect of long-term consumption of a probiotic bacterium, *Lactobacillus rhamnosus* GG, in milk on dental caries and caries risk in children [19].	2001	Randomized, double-blind, placebo-controlled intervention study	Children (1–6 years) N = 594	Less dental caries, lower *Streptococcus mutans* count, lower caries risk.
Taipale T et al.	*Bifidobacterium animalis* subsp. lactis BB-12 administration in early childhood: a randomized clinical trial of effects on oral colonization by mutans streptococci and the probiotic [43].	2012	Double-blind, placebo-controlled study	Newborns (1–2 months at baseline, followed up to 2 years) N = 106	Low MS colonization in children at 2 years, BB-12 has not permanently colonized the oral cavity.
Taipale T et al.	Administration of *Bifidobacterium animalis* subsp. lactis BB-12 in early childhood: a post-trial effect on caries occurrence at four years of age [44].	2013	Post-trial analysis of a randomized, double-blind, placebo-controlled study	Children (1–2 months at baseline, followed up to 4 years) N = 106	No difference in the occurrence of caries at 4 years.
Mayta-Tovalino F et al.	Efficacy of Probiotic Consumption on Oral Outcomes in Children and/or Adolescents: A Meta-Analysis [37].	2024	Meta-analysis of randomized controlled trials (RCTs)	Children and adolescents (various ages) N = 2622 (in 19 RCTs)	Probiotics likely reduce *Streptococcus mutans* counts, no significant effect on dental caries or other outcomes.
Kavitha M et al.	Evaluation of the efficacy of plaque reduction and gingival health among 6–12 years old school children before and after a short term daily intake of probiotic lozenge—A comparative study [38].	2022	Comparative study	Children (6–12 anni) N = 60	Significantly reduced plaque scores and improved gum health with probiotic tablets.
Alanzi A et al.	Effect of *Lactobacillus rhamnosus* and *Bifidobacterium lactis* on gingival health, dental plaque, and periodontopathogens in adolescents: a randomised placebo-controlled clinical trial [45].	2018	Randomized placebo-controlled clinical trial	Adolescents (13–15 years) N = 108	Significant reduction in gingival index, reduction in A. actinomycetemcomitans, *F. nucleatum* and *P. gingivalis*.
Dixit A et al.	Comparative evaluation of antimicrobial efficacy of various intracanal medicament in young permanent teeth: An in vivo study [20].	2024	In vivo study	Children (12–17 years) N = 30	Probiotics showed antimicrobial efficacy comparable to triantibiotic paste against *E. faecalis*.

## Data Availability

The data presented in this study are available upon reasonable request, after the signature of a formal data sharing agreement in anonymous form, from the corresponding author, because they are protected by privacy.
